# Leadership in strategic information (LSI) building skilled public health capacity in Ethiopia

**DOI:** 10.1186/1756-0500-4-292

**Published:** 2011-08-12

**Authors:** Italia V Rolle, Irum Zaidi, Jennifer Scharff, Donna Jones, Aynalem Firew, Fikre Enquselassie, Ashenafi Negash, Negussie Deyessa, Getnet Mitike, Nadine Sunderland, Peter Nsubuga

**Affiliations:** 1 Division of Public Health Systems and Workforce Development, Center for Global Health, Centers for Disease Control and Prevention, Atlanta, Georgia, USA; 2 Division of Global AIDS, Center for Global Health, Centers for Disease Control and Prevention, Atlanta, Georgia, USA; 3 Ethiopian Public Health Association, Addis Ababa, Ethiopia; 4 School of Public Health, Addis Ababa University, Addis Ababa, Ethiopia

## Abstract

**Background:**

In many developing countries, including Ethiopia, few have the skills to use data for effective decision making in public health. To address this need, the U.S. Centers for Disease Control and Prevention (CDC), in collaboration with two local Ethiopian organizations, developed a year long Leadership in Strategic Information (LSI) course to train government employees working in HIV to use data from strategic information sources. A process evaluation of the LSI course examined the impact of the training on trainees' skills and the strengths and weaknesses of the course. The evaluation consisted of surveys and focus groups.

**Findings:**

Trainees' skill sets increased in descriptive and analytic epidemiology, surveillance, and monitoring and evaluation (M and E). Data from the evaluation indicated that the course structure and the M and E module required revision in order to improve outcomes. Additionally, the first cohort had a high attrition rate. Overall, trainees and key stakeholders viewed LSI as important in building skilled capacity in public health in Ethiopia.

**Conclusion:**

The evaluation provided constructive insight in modifying the course to improve retention and better address trainees' learning needs. Subsequent course attrition rates decreased as a result of changes made based on evaluation findings.

## Background

The United States (U.S.) President's Emergency Plan for AIDS Relief (PEPFAR), implemented in 2003, is a significant undertaking by the U.S. government to prevent and treat HIV-infected persons in developing countries [1]. Strategic Information (SI) is an essential element of PEPFAR that ensures quality data are used to guide programs supported by this initiative. Surveillance, monitoring and evaluation (M and E), health management information systems, planning, and reporting are the core components of SI [2]. As the second cycle of PEPFAR broadens its focus to health systems strengthening in addition to scaling up services for HIV care treatment and prevention (PEPFAR I), the effective use of SI is key for this venture to be successful. A recent review of PEPFAR I by the Institute of Medicine (IOM) supports the role of SI in HIV-related activities. The IOM report recommended that as PEPFAR goes forward there is a need for quality data to guide interventions, evidence-based decision making, and ongoing evaluations and research [2].

The Centers for Disease Control and Prevention (CDC) has extensive experience in teaching the use of data for effective decision making using an applied approach that entails hands on practical training [3-5]. The use of data is central for evidence-based decisions as it leads to stronger and more appropriate responses to public health problems such as HIV. Ethiopia, located in the Horn of Africa, has a limited number of persons with skills to use data to assist with decision making in regards to HIV and other health related problems. Ethiopia is one of PEPFAR's first 15 focus countries and receives a substantial amount of funding for HIV activities through this initiative [6]. The prevalence of HIV in Ethiopia is 2.1% among adults aged 15-49 years and the epidemic varies from region to region [7,8]. Urban areas have a higher prevalence of HIV (7.7%) than rural areas (0.9%) [7,8]. To address Ethiopia's shortage of skilled public health staff, CDC collaborated with a local university and a local non-governmental organization (NGO) to conduct the first Leadership in Strategic Information (LSI) course for Ethiopia 2006-2007. Two of CDC's existing programs, the Field Epidemiology Training Programs [9-13], and Data for Decision Making short course [5], served as a model for LSI.

The overall goal of LSI is to improve the public health capacity of the Ethiopian health sector to use quality data to inform its decision making from SI sources. The training aims to equip trainees with the ability to use data to improve assessment, planning, surveillance, and M and E of HIV activities and other health problems in their respective regions. Five modules extended over a one year period to meet course objectives: HIV interventions and situational analysis (1 week), descriptive epidemiology (1 week), analytic epidemiology (2 weeks), HIV surveillance (2 weeks), and M and E (1 week). Each module consisted of sessions designed to teach specific instructional goals. Surveillance officers, public health laboratory technicians, and project managers from the regional HIV/AIDS Prevention and Control (HAPCO) department attended the course.

The trainees formed regional teams of four, based on job description and geographic location, to complete an applied learning project that is developed incrementally throughout the course using skills obtained from each of the modules. The regional group projects were an important element in the LSI training as they allowed trainees to apply knowledge learned in LSI directly to the workplace. These projects included a needs assessment as each group had to indicate which gaps in public health the projects would address; and also identify benefits this work would have in their regions. Each group gave an oral presentation that was scored by an expert panel consisting of five local public health professionals and an epidemiologist from CDC Atlanta. This score functioned as an indicator to show mastery of skills taught throughout the course. Lecturers from a local university's School of Public Health and personnel from CDC Atlanta facilitated the course. Lecturers from the local university served as mentors to the regional teams. The anticipated end result of the LSI training is an improvement in the skill set of trainees in using data for effective decision making from SI sources.

The purposes of this article are (1) to describe the results of an evaluation that examined the impact of the LSI training on trainees' skills and (2) to determine the strengths and weaknesses of the LSI structure to improve the course for the next cohort. The authors wish to demonstrate the utility of process evaluation early in course implementation to improve course outcomes and sustainability.

## Methods

**Figure 1:**
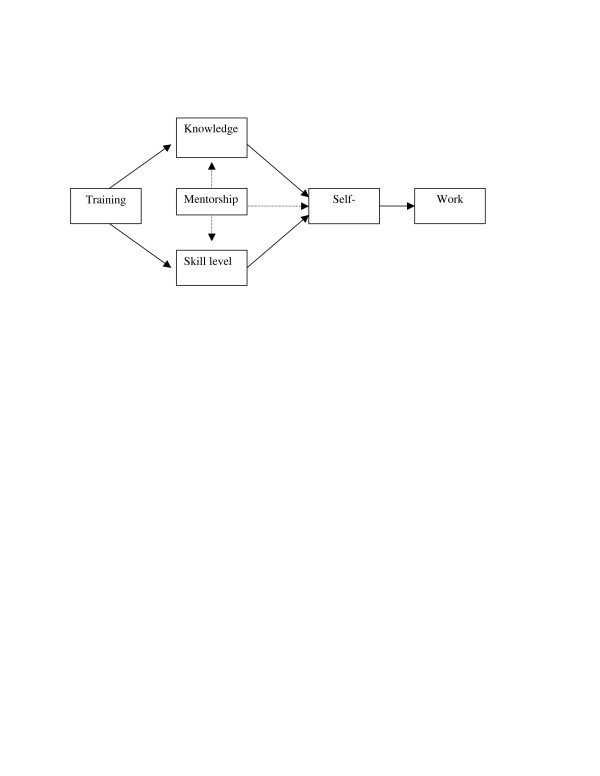
**LSI Evaluation Training Model**. Framework for evaluating training.

We adapted the "Model of Impact of Training on Maternal and Child Health Professionals" for the LSI evaluation [14,15]. The original model, developed by Farel et al., was used to examine how an online internet course assisted in improving data analytic skills of persons working in the field of maternal and child health (14). The LSI course although taught primarily in the classroom was similar to the maternal and child health online course as it also focused on teaching data analysis, and basic epidemiology and statistics to public health professionals. The adapted model outlines the key mechanisms through which the LSI training assists trainees to use data for decision making. According to the model (Figure 1), the training provided through LSI adds to the knowledge and skill level of the trainees. The box labelled "Training" includes lectures, exercises, assignments, and applied projects. The training activities contribute to trainees gaining new knowledge and skills and/or improving their skills in HIV interventions and situational analysis, descriptive epidemiology, analytic epidemiology, HIV surveillance, and M and E. Mentorship influences both the knowledge and skill level of trainees as the mentor assists trainees in applying the knowledge they learned. In addition to an improvement in knowledge and skill level, mentorship, allows trainees to become competent and confident in applying the information taught in the LSI course. This leads to self-efficacy, which is reflected in the work setting through successful practice of the knowledge and skills gained. Additionally, the work setting will improve for trainees as they become better equipped and skilled to handle their assigned duties related to using data sources.

The objectives of the LSI evaluation were:

(1) to determine if trainees applied the knowledge and skills learned during module training (e.g., carrying out a situational analysis, proper application of descriptive and analytical epidemiology, HIV surveillance, and performing M and E) on the job and in regional group projects;

(2) to examine trainees' opinions of the course;

(3) to ascertain how the LSI training is regarded by key stakeholders; and

(4) to determine its role in the context of increasing skilled public health capacity in Ethiopia.

The sample for the LSI evaluation included 15 trainees and six key stakeholders from the regional Ministry of Health (MOH), federal and regional HAPCO, WHO, and local universities. The evaluation was two-tiered and consisted of both quantitative and qualitative methods. The first component consisted of the trainees completing surveys after each module and the second component included an overall course survey after completion of the last module. Following each module, trainees gave their opinion of that module and raised any issues that needed to be addressed before the next module began. The overall course survey, in addition to asking trainees' opinion of the course as a whole, included several sections on application of knowledge and skills. These sections included: (1) previous training in HIV and epidemiologic methods; (2) relevance of skills obtained from LSI to the trainees' jobs; (3) retrospective pre- and post-training skills self-assessment conducted after the completion of LSI; and (4) rapport with their mentors and team members. Results from the overall course survey are presented, and are similar to those conducted at the end of each module. One focus group discussion with 10 trainees provided a forum for trainees to elaborate on responses provided in course surveys. The trainee focus group used a semi-structured questionnaire guide based on questions from the overall course survey.

For those trainees that withdrew from the course, the local NGO partnering with CDC emailed an electronic survey requesting their opinion of the modules they had attended and reasons for withdrawing. Key stakeholders provided feedback through a focus group discussion to determine how LSI contributes to HIV public health practice in Ethiopia. Six key stakeholders participated in the discussion. A semi-structured questionnaire guide was used to facilitate the discussion.

Data obtained from the module and overall course surveys were summarized using means and proportions. Major themes using the grounded-theory approach, a systematic approach with identification of major themes from text data, [16] were identified from the focus group discussions. The local NGO administered all interviews. All surveys and their results were anonymous.

## Results

The 23 LSI trainees came from regional health bureaus, with the exception of one, and held leadership positions such as head of department, team lead, or coordinator. Most (87%) of the trainees were men; only three women attended the course. Only 15 trainees received a certificate reflecting their full participation (all five modules completed) and two trainees (not considered members of LSI's first cohort) received a certificate for completing modules four (HIV Surveillance) and five (M and E). LSI had an overall completion rate of 65%. A drop of 25% occurred between the second and third module. The third module represented month five of the 12 month training schedule. Reasons for participants withdrawing from the course identified from the early withdrawal survey (n = 5) and the focus group (n = 10) included: the change from a postgraduate diploma to a certificate upon completion of the course; shortage of personnel in trainees' offices; length of course; pursuing graduate degrees outside Ethiopia; and involvement in a large acute watery diarrhea outbreak occurring at the same time as LSI.

The LSI trainees who completed the overall course survey (n = 11) had some prior exposure to epidemiology, surveillance, and use of statistical software (Table 1). At least half of the trainees reported previous training in HIV interventions, descriptive and analytic epidemiology, and M and E. Trainees lacked any prior training in situational analysis, HIV surveillance and statistical packages. A retrospective pre- and post-training skills self-assessment conducted after the completion of LSI indicated that trainees' self-reported skill level had improved in the areas taught by LSI (Table 2). The trainees reported that these improvements in performance were a result of the LSI training because they: (1) gained new knowledge and skills; (2) effectively analyzed data; (3) developed and implemented an appropriate epidemiological design for their group projects; and (4) felt confident in carrying out their assigned job duties. A major theme arising from the participant focus group discussion was the lack of training in data analysis prior to LSI. Trainees said collectively in the focus group, as a result of LSI: *"We are able to collect, analyze, and interpret data and use the findings"*.

**Table 1:** Previous Training

Topic (n = 11)	Yes
HIV interventions	64%
HIV situational analysis	27%
Descriptive epidemiology	55%
Analytic epidemiology	55%
HIV surveillance*	40%
Monitoring and evaluation*	50%
Epi Info (statistical program)	18%
STATA (statistical program)	0%
SPSS (statistical program)	18%
Excel (statistical program)	27%
SAS (statistical program)	0%

*n = 10 not 11.

**Table 2:** Retrospective pre- and post skills self-assessment

Topic	Skill level before course (median)					Skill level after course (median)				
**HIV Interventions**	**Low**				**High**	**Low**				**High**
**Situational Analysis**	1	2	3	4	5	1	2	3	4	5
a. Characterize the HIV/AIDS situation in your region *	2					4				
b. Identify current HIV interventions and strategies in your region*	3					5				
c. Describe the key points of an effective oral presentation*	3					4				
d. Develop a study protocol*	3					4				

**Epidemiology Methods**										
e. Develop a study hypothesis*	2					4				
f. Design and conduct an epidemiologic study*	2					4				
g. Analyze and interpret epidemiologic data by person, place, and time*	2					4				
h. Describe and calculate an odds ratio from a 2 × 2 epidemiologic table*	2					4				
i. Use Epi Info to enter data*	1					4				
j. Use Epi Info to clean and analyze data (n = 10)	1					4				
k. Use Epi Info to report data*	1					3				
l. Create effective charts, graphs, and tables*	2					4				
m. Present data and findings from surveys, studies, and surveillance analyses (n = 10)	2					4				

**HIV Surveillance**										
n. Discuss the purpose and use of surveillance data *	3					5				
o. Compare active and passive surveillance systems*	3					4				
p. Evaluate a public health surveillance system (n = 10)	3					4				
q. Describe the existing HIV surveillance system in your region*	3					4				
r. Describe HIV sentinel surveillance*	3					5				
s. Describe the purpose of HIV case surveillance (n = 10)	3					5				
t. Describe the importance of STI surveillance in relation to HIV (n = 9)	3					5				

**Monitoring and Evaluation**										
u. Develop program goals, objectives, and indicators*	2					4				
v. Describe the monitoring and evaluation framework *	2					4				
w. Develop program logic models*	2					4				
x. Link objectives to monitoring and evaluation measures and methods* y. Develop a M and E plan for a HIV program in your region*	2 2					4 4				

* n = 11.

Four regional groups gave oral presentations based on their course project findings. The titles included: (1) "Assessment of socio-demographic and socio-economic factors influencing adherence to antiretroviral therapy (ART) in Harari"; (2) "Assessment of knowledge, satisfaction of clients with ART services and the implementation of the roadmap plan in Amhara region"; (3) "Assessment of barriers and concerns for providing and using prevention of mother to child transmission in Dire Dawa"; and (4) "Assessment of the characteristics of patients with sexually transmitted infections in Mekelle Town, Tigray region". The trainee focus group discussion revealed the trainees felt confident preparing proposals and conducting scientific projects. Each regional group chose to collect primary data rather than using existing (secondary) data. The judges on the expert panel concluded that trainees had learned critical skills based on the LSI training, such as developing a project protocol and hypothesis, conducting proper program planning, and knowing the importance of data collection and analysis. The judges also observed from the presentations that the trainees needed to refine their analyses and correct some inconsistencies (e.g., improper use of generalizability for qualitative findings and weak recommendations).

Of the 11 trainees that completed the overall course survey, the majority (92%) felt the course met their expectations (Table 3). All participants agreed that the course material was relevant to their jobs. When asked to identify additional groups that could benefit from LSI, the following groups were listed: health bureau employees such as planners and persons working in M and E; program office supervisors, HIV/AIDS program managers, statisticians; and all persons working in the health sector. Trainees were asked about the length of the course, as this issue came up during several of the modules. Trainees judged the HIV interventions/situational analysis, and descriptive epidemiology modules "just right in length" and the analytic epidemiology, and M and E modules "too short" (Table 4). The majority of the trainees (73%) rated the overall course to be "just right in length".

**Table 3:** Overall Course Feedback

Statement n = 11	Strongly agree	Agree	Undecided	Disagree	Strongly disagree
Before coming to the course, I was informed of the purpose of the course.	9%	73%	9%	9%	0%
The information covered in the course addressed knowledge and skills needed for my job.	55%	45%	0%	0%	0%
The course content was consistent with objectives given by instructors.	55%	45%	0%	0%	0%
The course provided practical examples.	45%	46%	9%	0%	0%
The course provided enough time for questions and discussion of topics.	18%	64%	0%	18%	0%
The course met my expectations.	46%	46%	0%	9%	0%

**Table 4:** Modules and Course Feedback

Module (n = 11)	Too short	Just right	Too long
HIV interventions	27%	73%	0%
Descriptive epidemiology	36%	64%	0%
Analytic epidemiology	64%	36%	0%
HIV surveillance	18%	82%	0%
Monitoring and evaluation	45%	55%	0%
Overall course	27%	73%	0%

During the focus group discussion, trainees reported that the modules were useful, although they considered Module 1 (HIV interventions/situational analysis) to be basic and they believed Module 5 (M and E) required additional time for the complex concepts being taught. The recommendations provided by the trainees during the focus group included: (1) take into account length of LSI training when considering awarding a diploma or certificate; (2) grade trainees on an individual basis rather than by group; (3) revise and lengthen M and E module; (4) provide more time for statistical programming; and (5) use non-university affiliated mentors in addition to university mentors.

Three mentors completed a survey asking their opinion of their role in mentoring and their mentees (Table 5). Two of the mentors each had four trainees and the third mentor had seven trainees. When asked to rate their mentees, the majority of the trainees received a satisfactory to excellent score in regards to the following: commitment to learning, working on a team, requesting assistance when needed, maintaining contact throughout the course and applying the knowledge and skills from LSI to the team project. One criticism by the mentors was that 43% of trainees did not allocate enough time to work on the team projects.

**Table 5:** Mentor's rating of mentees

Statement	Poor	Fair	Satisfactory	Good	Excellent
Commitment to learning (n = 15)	0%	0%	13%	40%	47%
Working on a team (n = 12)	0%	0%	33%	58%	7%
Allocated enough time to work on project (n = 14)	43%	0%	14%	43%	0%
Requested assistance when needed (n = 12)	0%	8%	17%	17%	58%
Maintained contact throughout LSI (n = 13)	15%	0%	15%	32%	38%
Applied knowledge and skills from LSI to project (n = 12)	0%	8%	42%	42%	8%

Six key stakeholders representing three local universities, a regional health bureau, federal HAPCO, the WHO country office participated in a focus group discussion. The major themes resulting from the key stakeholders' focus group included: (1) LSI assists trainees in gaining valuable skills to become better program officers; (2) trainees had difficulty presenting qualitative research findings; (3) mentorship needs improvement; and, (4) the attrition rate needs to be investigated. The key stakeholders all concurred that LSI contributed to building skilled capacity in Ethiopia; however, the issues listed above needed to be addressed before another cohort was selected.

## Discussion

An unmet need of current personnel with data analysis skills determined the selection of government employees for LSI. All LSI trainees except for one were regional MOH employees. Results suggest that trainees acquired the appropriate skill set in descriptive and analytic epidemiology, surveillance, and M and E to assist their regional health bureaus to use data effectively in guiding their HIV programs. Throughout the course, trainees responded that the information taught was applicable to their jobs and assisted them to be better equipped and confident to handle their job assignments. Some trainees reported that upon returning to work, they conducted their own trainings for colleagues incorporating LSI concepts. Opportunities such as in-service trainings for co-workers by LSI trainees reinforce information taught in LSI as the trainee teaches others. Such opportunities illustrate that training of one person can benefit others in the workplace as knowledge sharing in the importance of data for decision making occurs.

The evaluation revealed several limitations. First, trainees did not complete a pre-test before the training began, thus, we could not determine quantitatively how the trainees' knowledge had improved in HIV interventions, situational analysis, epidemiology, surveillance, and M and E. However, the evaluation included a retrospective pre- and post-training skill level self-assessment which showed that perceived skill level increased significantly for the trainees as a result of LSI. Despite this measure being used solely after the course was completed, there is research indicating it is a valid measure [14]. Second, only 11 of 15 trainees that completed LSI responded to the overall course survey, and only five of the eight persons that withdrew responded to an electronic survey asking their opinion of LSI and their reasons for withdrawing. Therefore, answers provided by these two groups may not be representative of all the persons that attended LSI and those that withdrew early. Third, there was an attrition rate of 35% that may be due in part to a shortage of public health professionals working in the regional HAPCO offices. Most supervisors in regional offices allowed trainees to attend the course, but, due to a shortage of public health personnel, trainees were required to balance the LSI course and their routine work assignments. Thus, a course of one year in length may have placed a burden on some regional offices while personnel attended the training modules and dedicated additional time to coursework between modules.

As a result of the evaluation, implementers modified the structure of LSI prior to the second cohort beginning in 2009. The changes included: length of five months instead of one year, use of secondary data rather than primary data in research projects, the use of both university affiliated and non-university affiliated applied public health mentors from the trainees' respective regions, better support from work supervisors to provide trainees with enough time to accomplish LSI assignments, pre-test, and a decrease in the number of modules from five to three (Module 1 = situational analysis and descriptive epidemiology, Module 2 = analytic epidemiology, Module 3 = HIV surveillance and M and E).

As a result of these changes, trainee retention has increased dramatically. Cohort two, which occurred from March through August of 2009, saw 87% (n = 15) of trainees follow the course to completion. Cohort three (September, 2009-January, 2010) had a completion rate of 92% (n = 24). The fourth cohort is currently in process. Trainees continue to develop applied projects throughout the entire length of the modified course and complete a knowledge-based pre-test at the beginning of each module and a post-test at the end of each module. Following successful completion of the course, each trainee receives a certificate.

## Conclusion

As Ethiopia seeks to control its HIV epidemic, the importance of skilled human resource capacity in SI is essential. The newly acquired skills by LSI trainees in SI may assist PEPFAR in achieving its objectives as it focuses on health systems strengthening. PEPFAR and other HIV global health initiatives at present have made limited progress in assisting developing countries with strengthening their health systems [17]. This is perhaps due in part to the emergency nature of these initiatives as they sought to quickly control the HIV epidemic in the developing world. As the focus shifts from an emergency situation to a greater emphasis on building capacity, SI will play an important role in building and enhancing health systems. Each component of SI (surveillance, M and E, health management information systems, planning and reporting) will assist developing countries "to know their HIV epidemic" [18] and to be effective in using the second-generation of HIV surveillance that incorporates multiple existing and new data sources [i.e., behavioral and sexually transmitted infections surveillance] [19]. Further, LSI potentially can aid PEPFAR-funded countries with implementing the recommendations provided by the 2007 IOM assessment [2].

The process evaluation described in this paper was used to improve upon the LSI course. Due to changes made as a result of the evaluation, more trainees are able to successfully complete the course. Thus, LSI is better able to respond to the needs of the Ethiopian MOH as it works to prevent the spread of HIV/AIDS and provide services for those in need.

Next steps include validating LSI as a tool for building public health capacity to use data for effective decision making through further follow-up of graduates and their impact on the health care systems including HIV across Ethiopia. In conclusion, LSI contributes to building skilled capacity in Ethiopia as evidenced from this evaluation of trainees and key stakeholders. We anticipate that the use of SI data sources will improve as additional government and non-government personnel receive the appropriate skills through courses like LSI that promote an applied approach.

## Abbreviations

CDC: Centers for Disease Control and Prevention; HAPCO: HIV/AIDS Prevention and Control (Division of the Ethiopian Ministry of Health charged with prevention and control of HIV and AIDS); LSI: Leadership in Strategic Information training course; M and E: Monitoring and evaluation; MOH: Ministry of Health; PEPFAR: President's Emergency Plan for AIDS Relief; SI: Strategic information; U.S: United States

## Competing interests

The authors declare that they have no competing interests.

## Authors' contributions

IR coordinated the course, developed the evaluation design, drafted, and edited the manuscript. IZ assisted with designing the HIV surveillance module for the course, data analysis, and edited the manuscript. JS aided in developing the course, coordinating it, developing tools for the evaluation, and revising and editing the manuscript. DJ assisted in teaching during the course, developing the tools for the evaluation and revising and editing the manuscript. AF assisted in developing and coordinating the course and reviewing the manuscript. FE taught in the course, assisted with refining the evaluation tools, and edited the manuscript. AN helped to develop and coordinate the course and reviewed the manuscript. ND coordinated and taught in the course and revised the manuscript. GM coordinated and taught in the course, assisted with refining the evaluation tools, and reviewed the manuscript. NS led the development of the course in Ethiopia and reviewed and edited the manuscript. PN led the development of the course and reviewed and edited the manuscript.

The authors read and approved the manuscript.
